# Non-invasive fractional flow reserve estimation using deep learning on intermediate left anterior descending coronary artery lesion angiography images

**DOI:** 10.1038/s41598-024-52360-5

**Published:** 2024-01-20

**Authors:** Farhad Arefinia, Mehrad Aria, Reza Rabiei, Azamossadat Hosseini, Ali Ghaemian, Arash Roshanpoor

**Affiliations:** 1 Department of Health Information Technology and Management, School of Allied Medical Sciences, Shahid Beheshti University of Medical Sciences, Tehran, Iran; 2 Cancer Research Center, Shahid Beheshti University of Medical Sciences, Tehran, Iran; 3 Department of Cardiology, Faculty of Medicine, Cardiovascular Research Center, Mazandaran University of Medical Sciences, Sari, Iran; 4 Department of Computer, Yadegar-e-Imam Khomeini (RAH), Islamic Azad University, Janat-Abad Branch, Tehran, Iran

**Keywords:** Machine learning, Cardiology

## Abstract

This study aimed to design an end-to-end deep learning model for estimating the value of fractional flow reserve (FFR) using angiography images to classify left anterior descending (LAD) branch angiography images with average stenosis between 50 and 70% into two categories: FFR > 80 and FFR ≤ 80. In this study 3625 images were extracted from 41 patients’ angiography films. Nine pre-trained convolutional neural networks (CNN), including DenseNet121, InceptionResNetV2, VGG16, VGG19, ResNet50V2, Xception, MobileNetV3Large, DenseNet201, and DenseNet169, were used to extract the features of images. DenseNet169 indicated higher performance compared to other networks. AUC, Accuracy, Sensitivity, Specificity, Precision, and F1-score of the proposed DenseNet169 network were 0.81, 0.81, 0.86, 0.75, 0.82, and 0.84, respectively. The deep learning-based method proposed in this study can non-invasively and consistently estimate FFR from angiographic images, offering significant clinical potential for diagnosing and treating coronary artery disease by combining anatomical and physiological parameters.

## Introduction

Cardiovascular diseases (CVD) are the leading cause of death worldwide^1^. These diseases have been a significant concern in recent decades^2^, with nearly 18.5 million people expected to die from cardiovascular disease in 2019 and deaths from these diseases predicted to reach 23.6 million by 2030^3^.

Coronary artery disease is the most common CVD, affecting over twenty million adults in the United States and accounting for almost one-third of all cardiovascular-related deaths^4^. This disease leads to plaque accumulation in the coronary arteries, called stenosis^5,6^. Stenosis can occur as a partial or complete blockage of the coronary arteries, resulting in reduced blood supply to heart tissue^7^. Narrowing or blockage of the coronary arteries can lead to severe symptoms such as angina pectoris and even myocardial ischemia^8^.

Regarding the diagnosis of coronary artery disease, coronary angiography is considered the gold standard for evaluating the anatomical status of coronary arteries in patients^9^. Coronary angiography is an essential diagnostic tool for coronary artery disease, and the cardiologist's visual assessment of angiography images is used to identify narrowing and guide treatment^10^. However, visual evaluation of angiography images can lead to overestimating the severity of coronary artery stenosis^11^, and the variability in evaluation among evaluators makes it challenging^12–15^. On the other hand, visual evaluation is highly subjective and lacks accuracy, objectivity, and consistency^16^.

Although coronary angiography is a valuable method for describing the extent and severity of coronary artery disease, evidence shows that anatomical stenosis of the coronary arteries does not necessarily indicate the presence of myocardial ischemia, and the functional severity of coronary artery stenosis is the leading cause of myocardial ischemia^17,18^ The physiological assessment uses the fractional flow reserve (FFR) method, using a pressure wire to measure blood flow and pressure after passing through a stenosis following an agent such as adenosine injection. The results are displayed on a monitor along with the FFR value^19^. Based on extensive clinical evidence, using FFR to select patients and appropriate lesions for treatment helps avoid unnecessary procedures, reduces medical costs, and improves clinical outcomes^20^.

Various studies have shown that FFR is the gold standard for evaluating physiological coronary artery stenosis and making decisions regarding coronary revascularization. If this value is greater than or equal to 80, medical treatment is performed, and if it is less than 80, stenting is performed^21–25^. Using coronary angiography images alone in treatment decisions is challenging due to the variability in assessments among observers^12–14^. Additionally, performing revascularization without sufficient evidence of ischemia has significant health and economic consequences^19,23^. Therefore, evaluating coronary artery physiology is essential for providing appropriate treatment plans^18^.

However, despite the recommendations of treatment guidelines, the use of FFR for diagnosing coronary artery disease is limited worldwide^26,27^. It may be due to complexity, high cost, and the invasive nature of this method^28^. Treatment decisions still rely on visual estimation of stenosis severity from angiographic images, indicating a discrepancy between clinical guidelines and current practice^29^. On the other hand, visual assessment of angiographic images leads to an overestimation of coronary artery stenosis severity^11^. Since physiological assessment of stenosis severity during coronary angiography affects decision-making regarding revascularization in 43% of cases, all cardiac catheterization laboratories (Cath labs) should be capable of measuring the FFR^17^. Coronary angiography-based FFR eliminates the complications of the invasive nature of FFR and displays the values of coronary artery FFR^30^. Using FFR along with the coronary artery anatomy could significantly improve the clinical outcomes of patients. However, physiological assessment using anatomical data is challenging, and validation is required to confirm the accuracy of these models^31^. Therefore, physiological assessment using non-invasive methods with the help of angiographic images, obtaining the value of FFR, is of interest, and angiography image-based software provides the possibility of evaluating coronary artery physiology^25^.

In the past three decades, artificial intelligence (AI) has been widely used to improve the diagnostic accuracy of clinical tools and for data-driven decision-making in cardiovascular diseases. Additionally, AI-based systems can facilitate decision-making by improving interpretation processes, inference, and diagnostic accuracy^28,32,33^. As a subfield of artificial intelligence, machine learning has a subfield called deep learning, describing algorithms that analyze data with a logical structure similar to human reasoning. Deep learning is a subfield of machine learning that uses multiple layers of linear transformations to process data. Deep learning is a rapidly evolving field with many applications in medical imaging. Deep learning algorithms can extract and learn raw features from image data without limitations on feature extraction. Therefore, deep learning can be an ideal solution^34^. Deep learning is highly suitable for medical image segmentation^35^. Convolutional neural networks (CNN) are one of the most famous deep learning-based networks.

CNN is an artificial neural network consisting of convolutional, pooling, and fully connected layers. It has many applications for automatically extracting rules and features from various data types. CNNs are extensively used for image processing^36^ and classifying medical images^37^. They are used to segment coronary vessels^9^ and classify and identify stenosis in vessels^36,38^ using angiography images. Using pre-trained CNN models to increase accuracy and effectively reduce training time is a common approach in artificial neural networks. This method is referred to as transfer learning^36^.

This research endeavors to develop an advanced diagnostic and therapeutic system utilizing artificial intelligence (AI) techniques to surmount the constraints associated with traditional methods like coronary angiography and Fractional Flow Reserve (FFR) in the identification and treatment of moderate coronary artery stenosis. More specifically, our investigation aims to fill the existing gaps in this domain by introducing an innovative, comprehensive, and automated system, driven by artificial intelligence. This system is designed to process angiography images as input, providing a determination of FFR as either greater or less than 80. By doing so, it seeks to address the limitations inherent in conventional approaches, ushering in the integration of AI capabilities into the realm of cardiovascular diagnostics, allowing for the direct estimation of FFR values from angiography images.

## Related works

Estimating FFR using AI methods has been an essential topic in recent years, as researchers have attempted to calculate FFR non-invasively. Various AI methods, including deep learning-based methods, machine learning-based methods, and a combination of them, have been used along with different imaging tools such as CCTV, OCT, XCA, and IVUS. Table 1 shows the studies conducted in this field^39^.

**Table 1:** Studies on estimating FFR using AI methods^39^.

Reference (Year)	Modality	Number of patients/lesions	AI Methods	Prediction Task	Feature Engineering	Features	Performance
Hatfaludi et al.^40^	OCT	80/102 (LAD = 57, LCX = 20, RCA = 25)	DNN	Classification	Feature learning (DNN)	Anatomical OCT information	AUC = 0.763
							Accuracy = 0.775
							Sensitivity = 0.729
							Specificity = 0.815
							PPV = 0.778
							NPV = 0.772
Xue et al.^41^	CCTA	40/67(LAD = 32, D = 4, LCX = 10, OM = 1, RCA = 20)	BRNN	Regression	Feature learning (MLP)/ Handcrafted	Flow features	AUC = 0.95
	XCA					Radius features	Accuracy = 0.925
						Centerline Information	Sensitivity = 0.936
							Specificity = 0.881
							PPV = 0.8333
							NPV = 1
Lee et al.^42^	CCTA	144/200(LAD)	ANN, MLP	Classification	Feature learning (InceptionV3)/ Handcrafted	Morphological feature	Accuracy = 0.75 to 0.983
		Synthetic	RF, AdaBoost, SVM, GB, GP, KNN			Flow features	
						Biometric features	
Roguin et al.^43^	XCA	31(LAD = 25, LCX = 3, RCA = 3)	ANN	Regression	Feature learning	–	Accuracy = 0.9
							Sensitivity = 0.88
							Specificity = 0.93
							PPV = 0.94
							NPV = 0.87
Fossan et al.^44^	CCTA	50(LAD = 26, LCX = 13, RCA = 11)/150	FFNN	Classification	Handcrafted	Geometric features	Accuracy = 0.955
		(LAD = 78, LCX = 39, RCA = 33)			(VMTK)		Sensitivity = 0.94
							Specificity = 0.963
He et al.^45^	CCTA	60	SVM	Classification	Handcrafted (PyRadiomics)	left ventricular myocardial radiomics features	AUC = 0.8952
							Accuracy = 0.855
Cha et al.^46^	OCT	125(LAD)	RF	Classification	Handcrafted	OCT Geometric feature	AUC = 0.98
						Biometric features	Accuracy = 0.952
						Clinical features	Sensitivity = 1
							Specificity = 0.929
							PPV = 0.875
							NPV = 1
Kim et al.^47^	OCT	20	SVM	Classification	Handcrafted	Geometric feature	Accuracy = 0.75
	CCTA				(Boruta)	Flow features	Sensitivity = 0.5
						Biometric features	Specificity = 0.8
							PPV = 0.83
							NPV = 0.63
Gao et al.^48^	CCTA	180/13,000 Synthetic	RNN	Regression	Feature learning (RNN)	Centerline Information	AUC = 0.93
							Sensitivity = 0.84
							Specificity = 0.89
Carson et al.^49^	CCTA	25(LCA)	FFNN, LSTM, MPR	Regression	Handcrafted	Centerline Information	Accuracy = 0.72
					(VMTK)		Sensitivity = 0.9
							Specificity = 0.6
Kawasaki et al.^50^	CCTA	47/60	RF, LR, SVM	Classification	Handcrafted (CCTA Analysis)	Anatomic CCTA Descriptors	AUC = 0.698 to 0.835
						Functional Descriptors	
Kumamaru et al.^51^	CCTA	1052	NN	Classification	Feature learning (cGAN [Conditional Generative Adversarial Network])	-	AUC = 0.78
		(**131 labelled** LAD = 118, LCX = 49, RCA = 40))					Accuracy = 0.759
							Sensitivity = 0.846
							Specificity = 0.626
							PPV = 0.777
							NPV = 0.724
Zreik et al.^52^	CCTA	126/2340	CNN	Classification	Feature learning (CAE)	LVM Computed features	AUC = 0.74
						Centerline Information	Accuracy = 0.7
							Sensitivity = 0.7
							Specificity = 0.7
YIN et al.^53^	CCTA	13(LAD)	GPR	Regression	Handcrafted	Physiologic parameters	Sensitivity = 0.76 to 0.91
						Anatomic parameters	
Dey et al.^54^	CCTA	254/484	LB	Classification	Handcrafted	Patient factors	Accuracy = 0.8
					(AutoPlaque)	Quantitative CTA	Sensitivity = 0.73
							Specificity = 0.8
Zreik et al.^55^	CCTA	137/192(LAD = 104, LCX = 52, RCA = 36)	SVM	Classification	Feature learning (CAE)	Centerline Information	AUC = 0.87
							Accuracy = 0.8
Lee et al.^56^	IVUS	1328/1328(LAD = 891, LCX = 100, RCA = 337)	RF, SVM, ANN, LR,AdaBoost , CatBoost	Classification	Handcrafted	Computed IVUS features	Accuracy = 0.85 to 0.87
						Clinical variables	
						Patient factors	
						Quantitative CTA	
WANG et al.^57^	CCTA	63/71 (LAD = 32, LCX = 21, RCA = 18)	BRNN	Regression	Feature learning (MLNN [Multilevel Neural Network])	-	AUC = 0.664
							Accuracy = 0.873
							Sensitivity = 0.9714
							Specificity = 0.75
							PPV = 0.8293
							NPV = 0.9545
Denzinger et al.^58^	CCTA	95/345	GRU	Classification	Feature learning (RCNN [Recurrent Convolutional Neural Network]) / Handcrafted (PyRadiomics)	Radiomic features	AUC = 0.88
						Centerline Information	Accuracy = 0.87
							Sensitivity = 0.95
							Specificity = 0.61
							PPV = 0.9
							NPV = 0.74
Cho et al.^59^	XCA	1501/1501(LAD = 1017, LCX = 155, RCA = 329)	XGBoost	Classification	Handcrafted (CAAS-5)	Computed angiographic features	AUC = 0.87
						Clinical features	Accuracy = 0.81
							Sensitivity = 0.84
							Specificity = 0.89
							PPV = 0.77
							NPV = 0.79
Hamersvelt et al.^60^	CCTA	126	SVM	Classification	Feature learning (CAE [Convolutional Auto-Encoder])	LVM Computed features	AUC = 0.76
							Sensitivity = 0.846
							Specificity = 0.484
Hae et al.^61^	XCA	1132/1132(LAD = 718, LCX = 141, RCA = 273)	RF, SVM, LR, AdaBoost, CatBoost	Classification	Handcrafted (CAAS-5/ EchoPlaque 3.0)	Computed angiographic features	AUC = 0.84 to 0.91
	IVUS					Computed IVUS features	Accuracy = 0.78 to 0.84
						Clinical features	Sensitivity = 0.76 to 0.84
							Specificity = 0.8 to 0.85
							PPV = 0.63 to 0.71
							NPV = 0.88 to 0.92
Kim et al.^62^	IVUS	70/ 1447	XGBensmble, ANN, XGBoost, RF	Classification	Feature learning (VGG16)	Computed IVUS features	Accuracy = 0.73 to 0.81
						Patient factors	Recall = 0.63 to 0.71
							Precision = 0.61 to 0.74
							F1 score = 0.64 to 0.73
Zreik et al.^63^	CCTA	126	SVM	Classification	Feature learning (CAE)	LVM Computed features	AUC = 0.74
							Sensitivity = 0.71
Han et al.^64^	CCTA	252/408	AdaBoost	Classification	Handcrafted (SmartHeart)	LVM Computed features	Accuracy = 0.683
							Sensitivity = 0.527
							Specificity = 0.846
							PPV = 0.782
							NPV = 0.63
Itu et al.^65^	CCTA	87/125	DNN	Classification	Feature learning	Geometric features	AUC = 0.9
		(12,000 Synthetic)					Accuracy = 0.832
							Sensitivity = 0.816
							Specificity = 0.839
							PPV = 0.689
							NPV = 0.912

## Methods

This section consists of two parts. The first part includes the population, data structure, and data preparation methods. The second part examines the structure of the proposed method, data pre-processing methods, and the architecture of the proposed model discussed in detail below.

### Population

This retrospective cross-sectional study was conducted in 2023. The angiographic images of 41 patients who underwent angiography and FFR on the left anterior descending (LAD) coronary artery and were referred to a cardiac center between 2015 and 2022 were used in this study. Patients were referred for angiography based on symptoms such as chest pain or shortness of breath, as well as risk factors like family history, smoking, high cholesterol, etc., suggesting a preliminary diagnosis of coronary artery disease. Angiography was requested for further evaluation based on clinical presentation and noninvasive testing such as stress testing. The study participants ages ranged from 42 to 57 years and 19 participants were female. The participants had no stenosis, coronary flow impairment, acute myocardial infarction, or history of open-heart surgery. FFR was performed to physiologically evaluate the lesions with a visual estimation of 50% and 70% of stenosis. The data were collected by reviewing the medical records and the angiography department's archive. All patients underwent coronary angiography through the femoral artery using a Judkins catheter and conventional imaging. Multiple physicians performed angiography in all cases, and Ultravist-370 (Schering, Berlin, Germany) was used as the contrast agent. The injection was done manually (6–8 ml of contrast agent per injection). Coronary pressure was measured using a 0.014-inch pressure wire (St. Jude Medical, USA). The wire was guided and calibrated using a guiding catheter and placed approximately three centimeters past the stenosis. Maximum hyperemia was induced by intravenous administration of adenosine (average dose of 120 µg).

All experimental protocols were approved by the Institutional Review Board of Shahid Beheshti University of Medical Sciences, with the approval code IR.SBMU.RETECH.REC.1401.665, and were performed in accordance with relevant guidelines and regulations. Informed consent was obtained from all subjects and/or their legal guardians.

#### Data structure

**Figure 1:**
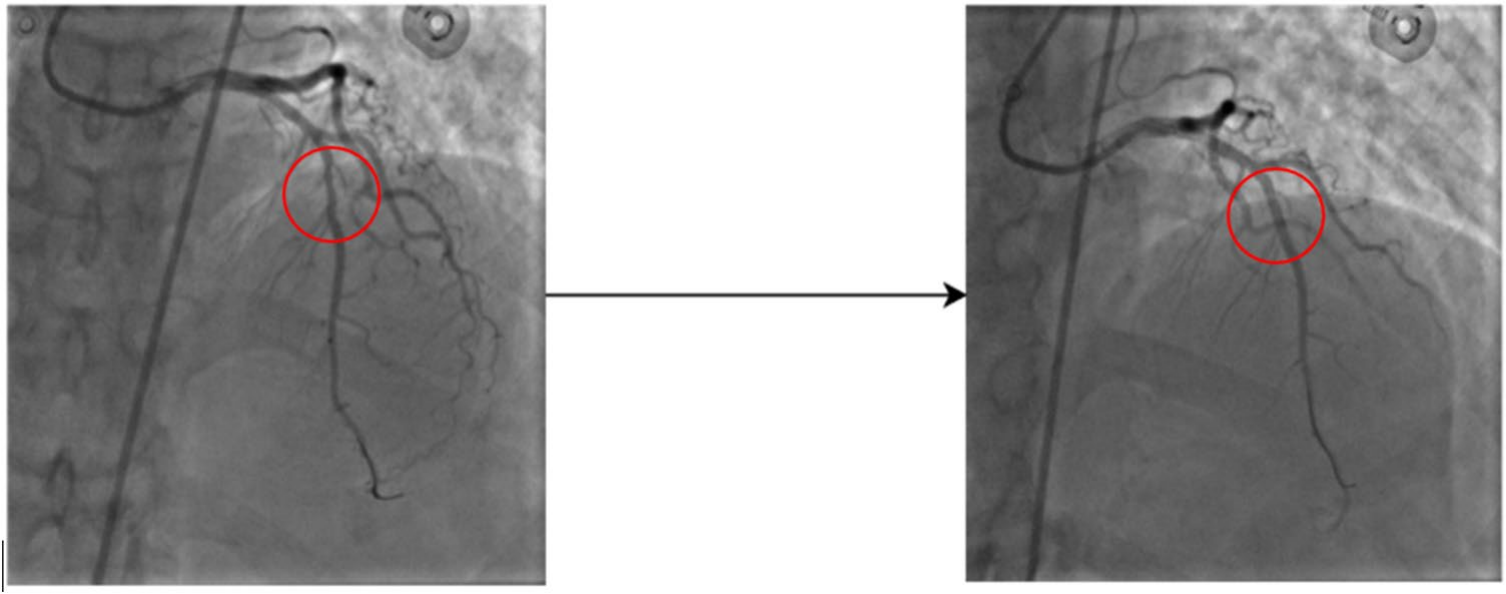
FFR Value before Revascularization is 0.8, and FFR Value after Revascularization is 0.9

The training data used in this study consisted of 2390 images from 18 patients before and after revascularization (All of these patients underwent FFR procedure after revascularization surgery, and their FFR values were greater than 80). Given that the arterial structure of a patient before and after revascularization surgery is the same, and the only difference is the removal of stenosis and increase in flow at the site of the lesion, the angiography images of these patients before stenting were classified into the category of patients with FFR ≤ 80, and the images after revascularization surgery were classified into the category of patients with FFR > 80. Therefore, assuming that the proposed model is sensitive to these changes and learns the desired region of interest better, this category of images was selected as the training dataset. Additionally, for model evaluation, the test dataset consisted of 772 images from twenty-three patients, including 14 patients with FFR > 80 and nine patients with FFR ≤ 80, as described in Table 2. The before-and-after images of patients were not used in the test dataset, and the images in each category in this dataset only included unique images of unique patients to have a fair and unbiased evaluation of the model. Figure 1 shows a patient's FFR value before and after revascularization surgery and changes in the region of interest (ROI) indicated with a red circle in the image.

**Table 2:** The dataset used for training and testing the proposed model.

	Train Set	Test Set	Total
No. patients	18	23	41
No. Images	2390	772	3625

#### Data preparation

An interventional cardiologist evaluated the angiography films of patients, and a total of 3625 black and white images related to the LAD artery from forty-one patients were included in the study, each measuring 512 × 512 pixels. This study classified patients into FFRH class for FFR > 80 and FFRL class for FFR ≤ 80.

### Proposed method

**Figure 2:**
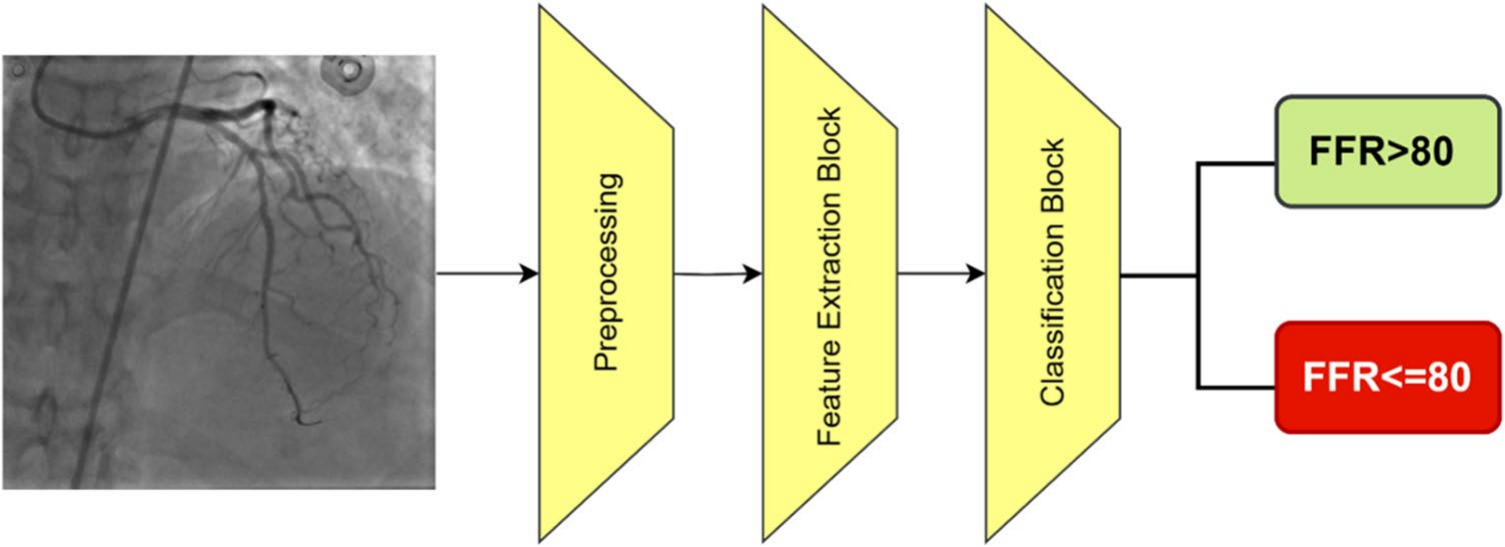
The overall structure of the proposed method.

Figure 2 illustrates the structure of the proposed method. First, pre-processing was performed on the input images, including decoding, resizing, normalization, augmentation, and histogram equalization. Then, the feature extractor inserted the obtained feature vector into the classifier block, and finally, the images were divided into two classes: FFR > 80 and FFR ≤ 80.

#### Preprocessing

Pre-processing is an essential step in deep learning that involves transforming and preparing raw data for effective utilization by a neural network^66^. It involves various techniques such as decoding, resizing, normalization, augmentation, and histogram equalization.

##### Decoding

Image decoding is converting the encoded image back to an uncompressed bitmap. The attribute channels indicate the decoded image's desired number of color channels.

##### Resizing

The image size of 380 × 380 pixels was selected using Grid search.

##### Data normalization

Normalization was applied to all images before entering the network.

The data were normalized to reduce the effect of intensity variations between radiographs. Normalization involves scaling the pixel values of images to a standard range or mean and unit variance to reduce the impact of varying lighting conditions on the image. Scaling involves rescaling the data to have similar units so that no feature dominates another^67^.

For data normalization, first, the pixel‐level global mean and standard deviation (SD) were calculated for all the images; next, the data were normalized using Eq. 1 where μ is the global mean of the image set X, σ is the SD, ε = 1e − 10 is an insignificant value to prevent the denominator from turning zero, i = [1 − 2083] is the index of each training sample, and Z_i_ is the normalized version of X_i_ (41).

1 $$Zi = \frac{Xi = \mu }{{\sigma + \varepsilon }}$$

##### Augmentation

Data augmentation is essential in deep learning models. It involves generalizing the training samples by transforming images without losing their semantic and intrinsic information. These transformations were randomly applied to the data^68,69^.

Data augmentation involves creating more training examples by transforming existing images through rotation, translation, contrast change, and zooming techniques.

Table 3 shows data augmentation techniques and the parameters used in this study.

**Table 3:** Details on the data augmentation techniques and parameters.

Type	Parameters
Random rotation	[-%30, + %30]
Random translation	[-%15, + %15]
Random zoom	[0, + %15]
Random contrast	[-%15, + %15]

##### Histogram equalization

The histogram information was used, and the most common intensity values were dispersed to produce a contrast-improved image^70^. Histogram equalization was performed using Eq. 2, where L is the maximum intensity level of the image; *M*: is the width of the image; *N*: is the height of the image; *N*: is the frequency corresponding to each intensity level; *r*_*j*_: the range of values from 0 to L-1; *P*_in_: the total frequency that corresponds to a specific value of *r*_*j*_; Rk: the new frequencies; *S*_*k*_: The new equalized histogram; where *k* = 0,1,2, ……, L − 113.

2 $$S_{k} = T (R_{k} ) = \mathop \sum \limits_{j = 0}^{k} P_{in} \left( {r_{j} } \right) = \frac{{\left( {L - 1} \right)}}{MN} \mathop \sum \limits_{j = 0}^{k} n_{j}$$

**Figure 3:**
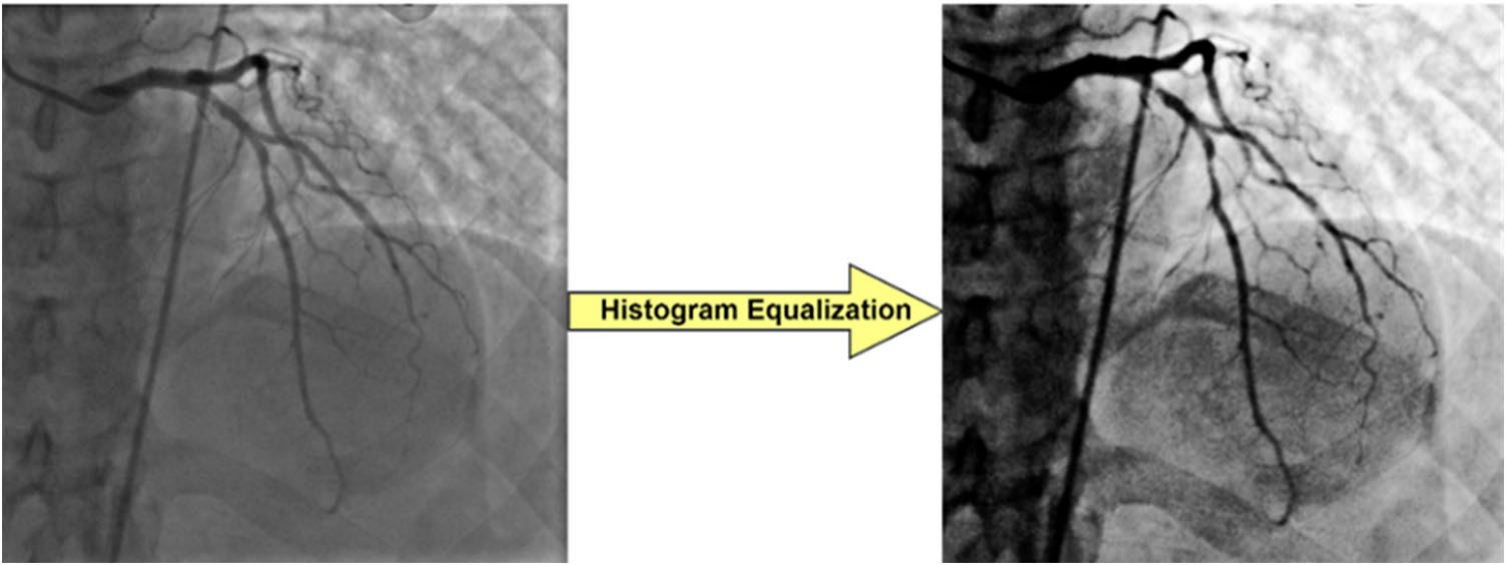
X-ray image before and after histogram equalization.

This study used this technique to adjust the contrast of the input image. Figure 3 shows an example of using this technique.

#### Model architecture

The proposed model consisted of feature extraction and classification blocks, explained in the following.

##### Feature extractor

**Figure 4:**
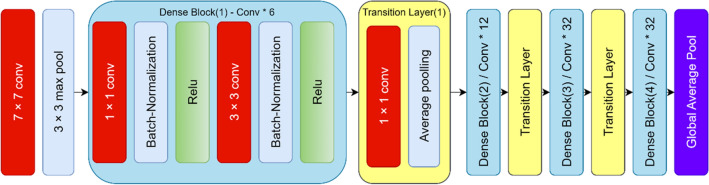
DenseNet‐169 architecture‐based feature extraction block^71^.

Nine famous pre-trained CNNs were used for image feature extraction, including DenseNet121^71^, InceptionResNetV2^72^, VGG16^73^, VGG19^73^, ResNet50V2^74^, Xception^75^, DenseNet201^71^, DenseNet169^71^, and MobileNetV3Large^76^. After running these networks on the dataset and evaluating them, DenseNet169 showed the best performance. This architecture consists of a convolutional layer, a pooling layer, four dense blocks, and three transition layers. the 4 dense blocks and 3 transition layers have been delineated separately using distinct boxes to showcase the individual components. For each dense block, the number of constituent layers is also indicated. For instance, Dense Block 1 is composed of 6 layers, with each layer utilizing batch normalization (BN), ReLU activation, followed by 1 × 1 and 3 × 3 convolutional filters of size 64. The subsequent Dense Blocks 2, 3 and 4 progressively increase the layers, while maintaining an identical structure of batch normalization, ReLU activation, and convolutional filtering. Finally, the transition layers in between the dense blocks employ batch normalization, ReLU activation, and 1 × 1 convolutions with 128, 256, and 512 filters respectively. We believe these model architecture clarifications provide improved understanding of the underlying DenseNet169 infrastructure per the reviewer’s suggestion. Please advise if further explanation or modification would be beneficial. Figure 4 illustrates the overall architecture of this network^71^.

##### Classifier

**Figure 5:**
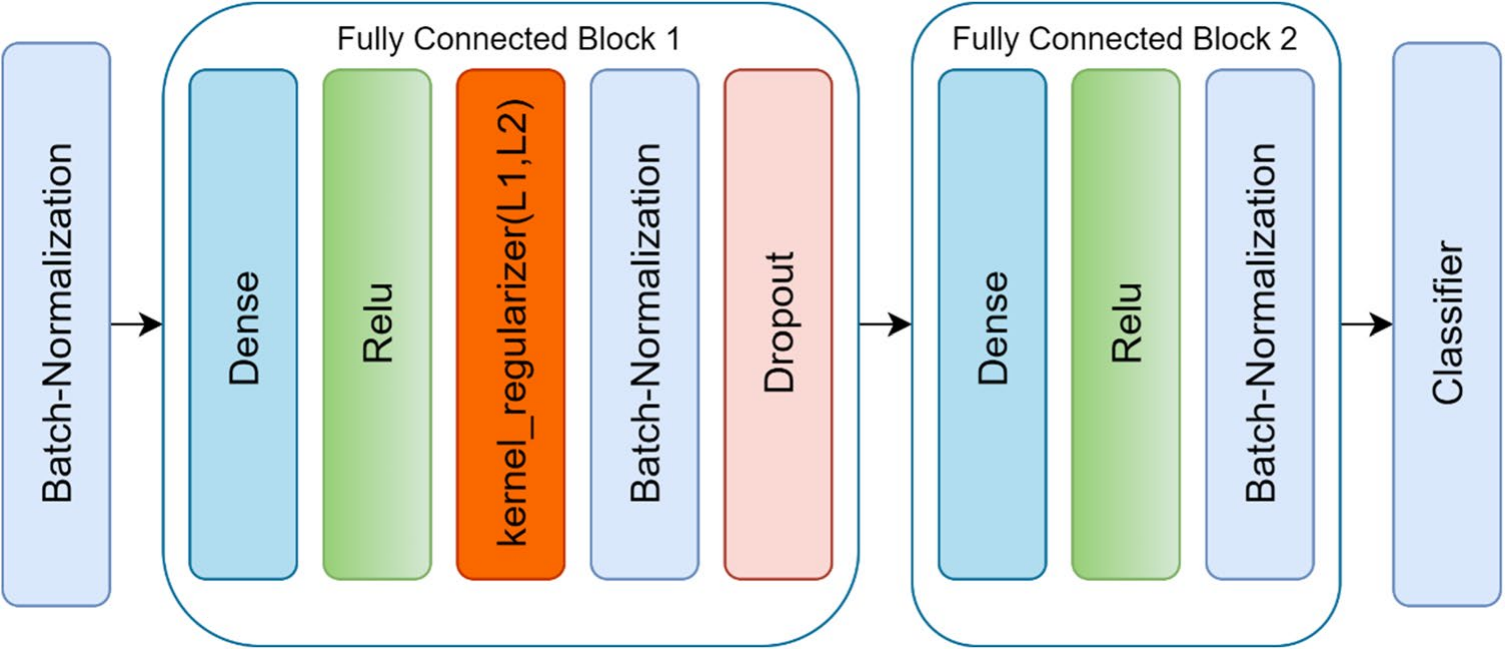
Classification block.

For classifying angiography images into two classes of FFR > 80 and FFR ≤ 80, a classifier block was designed, as shown in Fig. 5, in which two fully connected sequential blocks were used after the batch-normalization layer.

**Figure 6:**
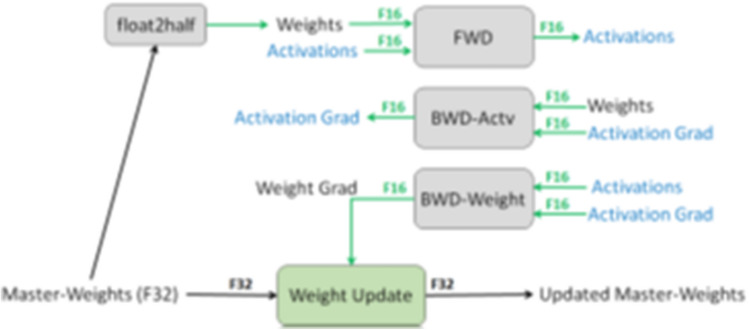
Mixed precision training iteration for a layer^83^.

The first block consisted of dense, ReLU, Kernel Regularizer L1L2, batch‐normalization, and dropout layers. The second block comprised dense, ReLU, and batch‐normalization layers, respectively. Figure 6 displays these steps in detail.

The classifier was a dense layer with two neurons, and the Softmax function was applied to these representations. This function specified the probability of allocating each sample to one out of Two classes, and its value fell in the [0,1] range. Figure 5 displays these steps in detail.

#### Training and implementation

The feature extractor block was completely frozen using the transfer learning approach in the first training phase and included non-trainable parameters. This model was trained for several epochs with weights obtained after fitting the ImageNet dataset. However, all parameters of the classifier block were trainable.

The first training phase used the Adam optimizer with an initial learning rate of 1e-2 and a decay rate of 1e-5. The Adam optimizer with an initial learning rate of 1e-4 and a decay rate of 1e-6 was used in the second training phase. In both training phases, cosine Annealing was used. In the second phase of fine-tuning, all network layers except for the first eight layers, the feature extractor, and the first convolutional block were trainable and frozen.

**Figure 7:**
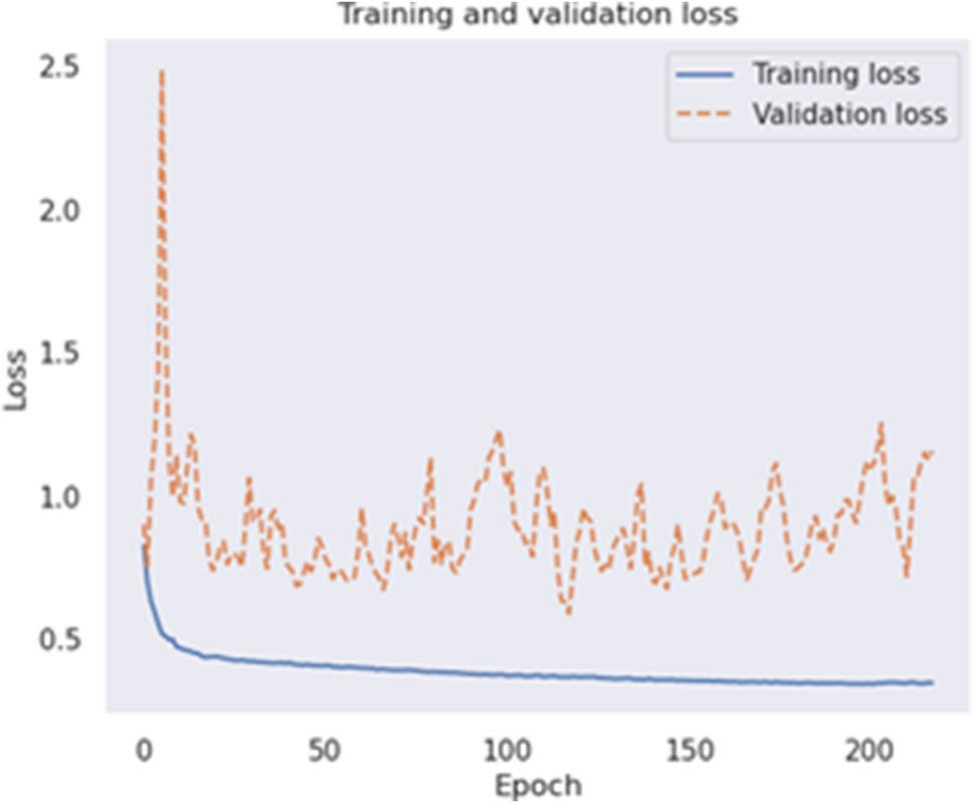
The loss of the proposed model during training. Model converged after 238 epochs.

The training process consisted of 120 epochs in the first phase and 600 in the second phase. Early stopping was considered at ten epochs in the first and 100 in the second phases. In the second phase of training, validation loss was also monitored. If it remained constant for ten epochs and did not improve, the learning rate would decline by 20%. Validation accuracy was also monitored, and only the model with the best weights obtained was saved. The optimal hyperparameter values were obtained using grid search. The value of the kernel regularizer parameters was l1 = 1e-5 and l2 = 1e-4. These architectures were implemented using Python language and the Keras library and executed on Google's TPU v3-8. Figure 7 shows the training and validation loss after 238 iterations during the training process.

##### Loss function

Cross-entropy was used as the loss function, which is a metric for measuring the performance of a classification model in machine learning and is defined by Eq. 3, Where *P*(*x*) is the probability of the event *x* in *P*, *Q*(*x*) is the probability of event *x* in *Q*, and the log is the base-2 logarithm^77^.

3 $$H\left( {P, Q} \right) = {-} sum\,x\,in X P\left( x \right) * log\left( {Q\left( x \right)} \right)$$

##### Learning rate schedule

The learning rate schedule is a pre-defined framework that adjusts the learning rate between epochs or iterations to avoid getting stuck in the local optimum as training progresses. This study used a warm restart cosine annealing for the learning rate scheduling program, considering the best weights as the restart points. It is demonstrated in the following equation (Eq. 4), where the best weights are considered as the restart points.

Within $i$-th run, the learning rate is decayed with a cosine annealing for each batch as follows:

$\eta_{min}^{i}$ and $\eta_{max}^{i}$ are ranges for the learning rate, and $Tcur$ accounts for how many epochs have been performed since the last restart. Since Tcur is updated at each batch iteration *t*, it can take discredited values such as 0.1 and 0.2. Thus, ${ }\eta t = \eta_{max}^{i}$ when $t = 0$ and $Tcur = 0$. Once $Tcur = { }Ti$, the ${\text{cos}}$ function will output − 1, so $\eta t = \eta_{min}^{i}$^78^.

4 $$\eta t = \eta_{min}^{i} + \frac{1}{2}\left( {\eta_{max}^{i} - \eta_{min}^{i} } \right)\left( {1 + \cos (\frac{Tcur}{{Ti}}\pi } \right)$$

##### Custom weighting

The unequal number of class samples, known as class imbalance, is an issue in machine learning classification problems. It affects the prediction model and leads to bias. Custom weighting was used to prevent this challenge, with a weight of 0.8 for the high-count class and 1.32 for the low-count class. These values represent the weighted average of the number of samples in each class.

##### Label smoothing

Label smoothing was used to improve the generalizability of the model.

Label smoothing is an effective regularization tool for deep neural networks (DNNs) and can implicitly calibrate the model's predictions. It significantly impacts the model interpretability and improves model calibration and beam search. It accounts for the possible mistakes in datasets, so maximizing the likelihood of $\log p\left( {y{|}x} \right)$ can be directly harmful. For a small constant ε, the training set label $y$ is correct with the probability of 1—ε and incorrect otherwise. Label Smoothing regularizes a model based on a Softmax with k output values by replacing the hard 0 and 1 classification targets with targets of $\frac{{{\varvec{\upvarepsilon}}}}{k - 1}$, respectively^76,79–81^.

##### Techniques to prevent overfitting

Overfitting is a fundamental problem in supervised machine learning, preventing models from perfectly generalizing to observed training data and unseen test set data. Overfitting occurs due to noise, limited training set size, and classifier complexity^82^. In order to address concerns related to potential overfitting in our model, several regularization techniques were strategically incorporated during the model development phase. Batch Normalization was applied to normalize the activations of various layers, enhancing the stability of the learning process. Additionally, Dropout with a rate of 0.2 was implemented on specific layers to introduce a level of randomness, preventing the model from relying too heavily on specific features present in the training set. Furthermore, L1L2 Kernel Regularizer was employed on the Dense layer with carefully chosen coefficients to penalize large weights and reduce model complexity. These regularization techniques collectively contribute to the robustness of our model by striking a balance between fitting the training data and generalizing well to new, unseen data. The effectiveness of these measures is evident in the model's performance, as illustrated in Fig. 7 and discussed in the results section.

##### Mixed precision

Mixed precision decreased fitting/training time and reduced memory usage during training. Figure 6 illustrates the mechanism of this method.

### Ethical approval

All experimental protocols were approved by the Institutional Review Board of Shahid Beheshti University of Medical Sciences, with the approval code IR.SBMU.RETECH.REC.1401.665, and informed consent was obtained from all subjects and/or their legal guardians.

## Experiments

In this section, the performance evaluation parameters of the model are first explained, then the proposed method's performance is evaluated, and the model training results are reported. Furthermore, various well-known pre-trained networks were also used, and their training results were compared with the proposed method.

### Evaluation metrics

Evaluation metrics are different types of measures to evaluate the performance of a deep learning model. They are mainly Accuracy (3), Precision (4), Recall (4), F-Measure (6), and Specificity. The number of true-positive (TP), false-positive (FP), true-negative (TN), and false-negative (FN) values are required to measure these parameters, as mentioned below.

5 $$Accuracy = \frac{TP + TN}{{TP + FP + FN + TN}}$$

6 $$Precision = \frac{TP}{{TP + FP}}$$

7 $$sensitivity\left( {Recall} \right) = \frac{TP}{{TP + FN}}$$

8 $$F1 - Score = \frac{{2 * \left( {Recall * Precision} \right)}}{Recall + Precision}$$

9 $$Specificity = \frac{TN}{{TP + FN}}$$

### Model evaluation

In this section, the evaluation results of the model on the test dataset were reported. For evaluating the proposed model, the cross-validation method was used. Cross-validation is a statistical method for evaluating and comparing learning algorithms by dividing the data into model training and validation^84–86^. The main form of cross-validation is k-fold cross-validation, where k equals the number of folds. This type of validation is performed as follows:

In each iteration, one or more learning algorithms use *k* = 1 folds of data to learn one or more models, and subsequently, the learned models are asked to make predictions about the data in the validation fold. The performance of each learning algorithm on each fold can be tracked using some predetermined performance metric like accuracy. Different methodologies, such as averaging, can be used to obtain an aggregate measure from these samples, or these samples can be used in a statistical hypothesis test to show that one algorithm is superior to another.

**Figure 8:**
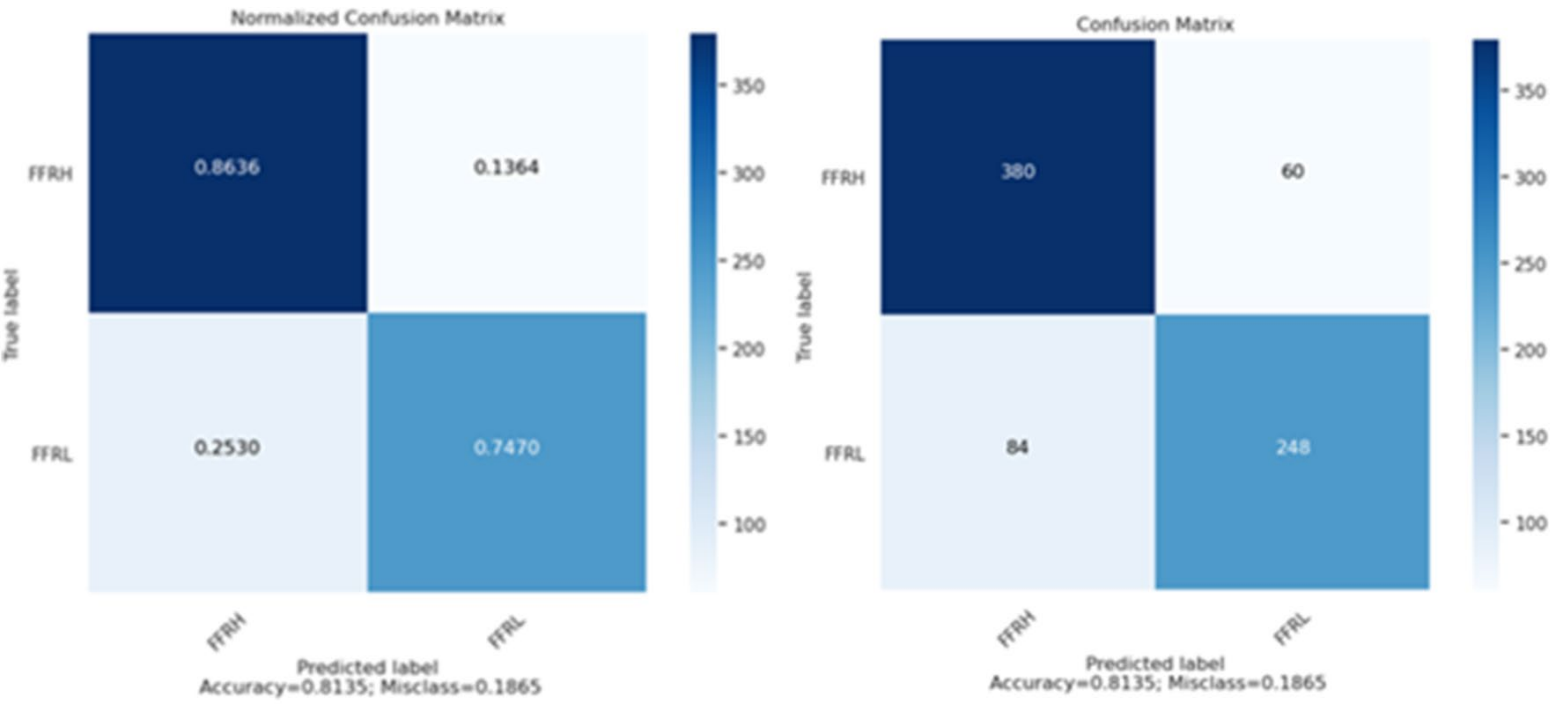
Confusion matrix of model evaluation on the test data set.

This study used five-fold cross-validation to validate the proposed model. The final results of evaluating the proposed model using this method are reported in Table 4 and Fig. 8.

**Table 4:** The proposed model's evaluation results in DenseNet-169 Network.

	Accuracy	Sensitivity	Specificity	Precision	F1-Score	Support
FFR > 80	0.81	0.86	0.75	0.82	0.84	440
FFR < = 80	0.81	0.75	0.86	0.81	0.77	332
Weighted avg	0.81	0.81	0.81	0.81	0.81	772

**Figure 9:**
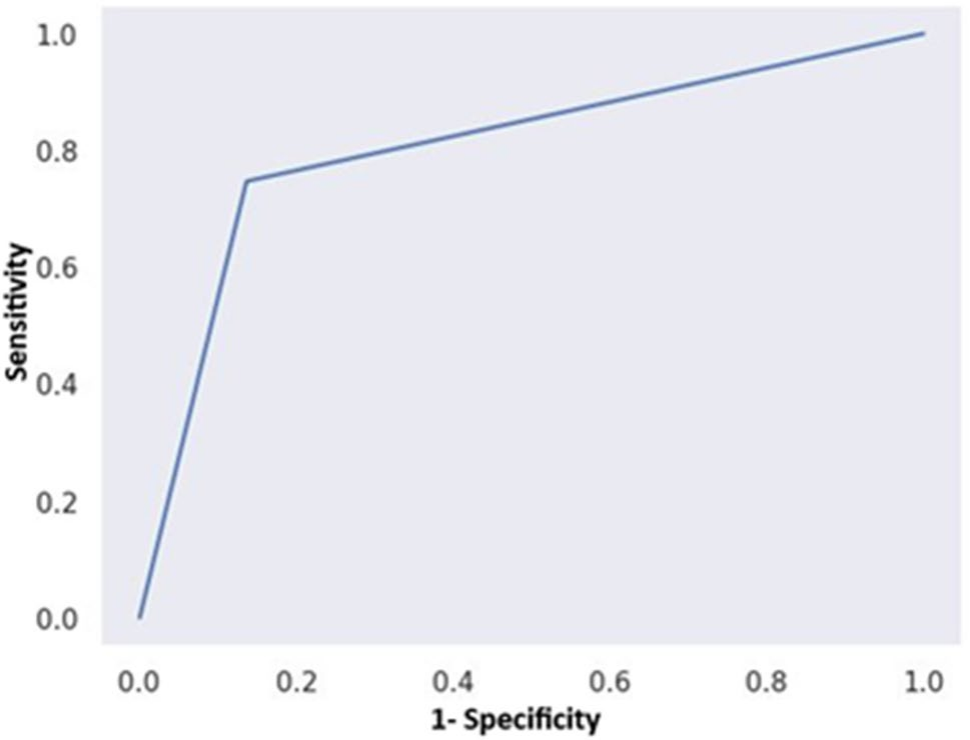
Fig. 1—Receiver Operating Characteristic (ROC) curve on the test data set.

The Receiver Operating Characteristic (ROC) curve in Fig. 9 illustrates the predictive model's performance for Fractional Flow Reserve (FFR) with an Area Under the Curve (AUC) of 0.81. This AUC value signifies a strong discriminatory capacity, effectively distinguishing between FFR > 80 and FFR < = 80 classes. Specifically, the model excels in discerning FFR > 80 and FFR < = 80 classes, as indicated by the AUC value. The 95% confidence interval for the AUC, [0.777, 0.833], ensures the precision of this discrimination. Moreover, the exceedingly low p-value (< 0.001) underscores the model's statistical significance, indicating a substantial and meaningful difference compared to the baseline value of 0.5.

### Review and comparison of pre-trained feature extractors

Nine pre-trained CNNs, including DenseNet121, InceptionResNetV2, VGG16, VGG19, ResNet50V2, Xception, MobileNetV3Large, DenseNet201, and DenseNet169 (Proposed), were used for image feature extraction and were evaluated with the test dataset. These models were compared based on the accuracy parameter. Table 5 shows the obtained results.

**Table 5:** Comparison of the prediction accuracy of the proposed model on the test set using different pre-trained networks as feature extractors.

Feature extractor	Accuracy
MobileNetV3Large	0.55
VGG16	0.57
VGG19	0.63
ResNet50V2	0.70
InceptionResNetV2	0.71
Xception	0.72
DenseNet201	0.74
DenseNet121	0.80
Proposed (DenseNet-169-Based)	0.81

The performance outcomes from assessing the three highest-accuracy models using the evaluation the test data are presented in Table 6.

**Table 6:** Evaluation results for top 3 pre-trained feature extractors.

Feature extractor	Class	Accuracy	sensitivity	Specificity	Precision	F1-Score	Support
DenseNet-169	FFR > 80	0.81	0.86	0.75	0.82	0.84	440
	FFR < = 80	0.81	0.75	0.86	0.81	0.77	332
	Weighted avg	0.81	0.81	0.81	0.81	0.81	772
DenseNet121	FFR > 80	0.80	0.81	0.80	0.84	0.83	440
	FFR < = 80	0.80	0.80	0.80	0.76	0.78	332
	Weighted avg	0.80	0.80	0.80	0.80	0.80	772
DenseNet201	FFR > 80	0.74	0.70	0.79	0.82	0.76	440
	FFR < = 80	0.74	0.79	0.70	0.67	0.72	332
	Weighted avg	0.74	0.74	0.74	0.74	0.74	772

## Discussion

In the present study, a fast, end-to-end, automated deep learning model was designed for estimating FFR values using angiography images. This model can classify angiography images into two classes, FFR > 80 and FFR < = 80, with no manual annotation and an overall accuracy of 81%. Multiple studies have shown a correlation between anatomical and physiological parameters^87,88^, and the current study's findings also provide further insights into how angiography features affect FFR values.

Although angiography is the gold standard for evaluating the severity of coronary lesions, physiological evaluation is the determining factor for treatment planning in patients with coronary artery disease^89^. FFR is considered the gold standard for the physiological assessment of coronary artery stenosis and is a strong indicator for diagnosis, treatment, and determining the approach for interventions. However, the invasive nature of FFR evaluation and its high cost has led to a lack of enthusiasm among healthcare professionals to use this method routinely in the Cath lab. The proposed method in this study has the potential to be used routinely in Cath labs due to its low cost, no need for additional data entry or extra workload for the cardiologist, online usability, and no need for changes in workflow in the Cath lab. However, this method requires external validation. External evaluation in deep learning checks a model's performance on new, distinct data, ensuring its generalization and minimizing overfitting for real-world applications^90–92^.

The present study shows that in recent years, significant efforts have been made to integrate anatomical and physiological parameters, indicating this method's clinical value for physicians and patients. However, integrating anatomical and physiological parameters is a significant challenge^93^. Various methods have been developed to calculate FFR without an invasive pressure wire or inducing hyperemia^31^. The present study's findings also demonstrate that image-based deep learning for determining FFR is a non-invasive and cost-effective method that can be used to match the visual and physiological features of coronary artery stenosis.

In recent years, an end-to-end framework has been introduced in deep learning, and its benefits in the health field have been investigated^94,95^. This study's proposed model demonstrates the advantages of using this approach for estimating FFR. Physicians can use this model to evaluate physiological conditions without entering additional data and manual annotation, only by inputting angiography images. Additionally, to facilitate the successful implementation of this method in Cath labs, systems based on this model can display FFR values online. On the other hand, the FAME study shows that only 35% of patients with stenosis between 50 and 70% are found to be significant stenosis in FFR evaluation. In other words, a model that can detect more insignificant stenosis will result in fewer unnecessary FFRs.

The existence of a non-invasive method for reducing unnecessary FFRs is also very important, and artificial intelligence, due to its non-invasiveness and the lack of need to change the workflow of the Cardiac catheterization laboratory, can be an excellent solution. This highlights the potential value of an accurate non-invasive AI-based FFR estimation approach. Such a method could help avoid unnecessary invasive FFR procedures and their associated costs and complications in cases where non-invasive assessment predicts non-significant stenosis. This is particularly relevant given that studies show only a subset of intermediate coronary lesions are found to be hemodynamically significant when measured invasively. More widespread adoption of validated non-invasive FFR estimation techniques may improve clinical workflows and benefit both patients and healthcare systems.

In the present study, the DenseNet169 model outperformed other models in detection of insignificant stenosis. Compared to other studies in this field, our proposed method requires only a single view from the angiography image with no need for annotation or additional parameters, without altering existing clinical workflows, yet still achieves state-of-the-art performance by utilizing a deep learning approach.

## Study limitations and future considerations

While our study provides valuable insights into FFR estimation using angiography images, it is essential to acknowledge certain limitations. Firstly, the relatively small sample size of 41 patients might impact the generalizability of our findings. Future research endeavors should prioritize the inclusion of a larger and more diverse cohort to enhance the robustness and external validity of the proposed model.Additionally, this study focused solely on the parameters present in angiography images, omitting potential influential factors such as age and gender. The exclusion of these variables may limit the comprehensive understanding of FFR estimation. Future investigations could explore the incorporation of additional clinical parameters to refine and expand the predictive capabilities of the model. External evaluation of our method on independent datasets will also be important to further validate the generalizability of our findings. External evaluation is something that will be a focus of our future work.

## Conclusion

This study designed an intelligent, fast, end-to-end, and automated method using the CNN architecture, the concept of transfer learning, and the pre-trained DenseNet169 network for estimating FFR values based on angiography images. This model can estimate FFR non-invasively with an overall accuracy of 81%. DL-based angiography image-derived FFR is a valuable tool for decision-making in diagnosing and treating stenosis in Cath labs. This model can assist cardiologists in decisions about diagnosis and treatment of moderate stenosis by combining physiological and anatomical parameters of coronary arteries.

## Data Availability

Due to the policies and guidelines of Shahid Beheshti University of Medical Science, data is not allowed for publication. The raw data supporting the conclusions of this article will be made available by the authors without undue reservation. The Python source codes used to develop the model are deposited on GitHub (https://github.com/MehradAria/FFR-Estimation).

## Abbreviations

A: Automatically
AI: Artificial intelligence
ANN: Artificial neural network
BRNN: Bidirectional multilayer recursive neural network
BRNN: Bidirectional multilayer recursive neural network
CAD: Coronary artery disease
CCTA: Coronary computed tomography angiography
cGAN: Conditional generative adversarial network
CVD: Cardiovascular diseases
DL: Deep learning
DNN: Deep neural networks
FFR: Fractional flow reserve
GB: Gradient boosting
GP: LogitBoost
GRU: Gated recurrent units
IVUS: Intravascular ultrasound
LAD: Left anterior descending artery
LCA: Left coronary artery
LCX: Left circumflex artery
LR: Logistic regression
LVM: Left ventricular myocardial
M: Manually
ML: Machine learning
MLNN: Multilevel neural network
MLP: Multilayer perceptron
OCT: Optical coherence tomography
RCA: Right coronary artery
RCNN: Recurrent convolutional neural network
RF: Random forest
SVM: Support vector machine
XCA: X-ray coronary angiography
